# Photogrammetry-Based Volume Measurement Framework for the Particle Density Estimation of LECA

**DOI:** 10.3390/ma15155388

**Published:** 2022-08-05

**Authors:** Karol Brzeziński, Adam Duda, Adam Styk, Tomasz Kowaluk

**Affiliations:** 1 Faculty of Civil Engineering, Warsaw University of Technology, Armii Ludowej Ave. 16, 00-637 Warsaw, Poland; 2 Faculty of Civil and Transport Engineering, Poznan University of Technology, Piotrowo 5, 60-965 Poznan, Poland; 3 Faculty of Mechatronics, Warsaw University of Technology, Andrzeja Boboli 8, 02-525 Warsaw, Poland

**Keywords:** photogrammetry, lightweight clay aggregate, particle density, volume measurement, computed tomography

## Abstract

This paper presents a photogrammetry-based volume measurement framework for the particle density estimation of Lightweight expanded clay aggregate (LECA). The results are compared with computed tomography (CT) and Archimedes’ method measurements. All of the steps required in order to apply the proposed approach are explained. Next, we discuss how the interpretation of open pores affects the results of volume measurements. We propose to process the shapes obtained from different methods by applying an Ambient Occlusion algorithm with the same threshold, *t* = 0.175. The difference between the CT and SfM methods is less than 0.006 g/cm^3^, proving that the photogrammetry-based approach is accurate enough. The Archimedes’ method significantly overestimates the density of the particles. Nevertheless, its accuracy is acceptable for most engineering purposes. Additionally, we evaluate the accuracy of shape reconstruction (in terms of the Hausdorff distance). For 95% of the grain’s surface, the maximum error is between 0.073 mm and 0.129 mm (depending on the grain shape). The presented approach is helpful for measuring the particle density of porous aggregates. The proposed methodology can be utilized in order to estimate intergranular porosity, which is valuable information for the calibration of DEM models.

## 1. Introduction

Lightweight expanded clay aggregate (LECA) is a lightweight and porous ceramic aggregate produced from clay with a high content of clay fraction fired during the rotary kiln process, at a temperature of approx. 1100–1200 °C [1]. During firing, the clay swells and forms tough, irregular, and porous aggregates with a sintered ceramic surface. Lightweight aggregates are used in the construction industry in order to produce a lightweight concrete and a thermal insulation layer for floors, ceilings, and roofs, including green roofs. In the case of the insulation of foundations and ground floors, they can also act as drainage. Due to its low bulk density, its high shear strength, and its excellent water permeability, LECA is widely used in geotechnics. It is used to construct lightweight embankments on weak ground, as backfill for bridge abutments and road culverts, as drainage, and for reducing the weight of various pavement structures, e.g., sports fields and car parks.

The compaction of the soil medium is directly related to the porosity of the skeleton. Compaction affects the mechanical properties of soils, including shear strength or soil compressibility. In geotechnical applications, the currently used measure of LECA compaction is the reduction of the initial volume of the backfill material during its compaction (usually up to 10% [2]). It is difficult to use other measures of compaction, such as the void ratio, due to the difficulties in determining the particle density of the porous grains. Therefore, an easy and reliable method of determining the particle density would allow to control the compaction of LECA backfill using other methods (e.g., the void ratio).

Information about the particle density of grains may also be helpful for the calibration of numerical models using the DEM method [3], which consists of the explicit modeling of the granularity of the medium. If the material composition is well represented (in terms of relative density, particle shape, and size), then simple constitutive laws are sufficient in order to reproduce the mechanical properties of a granular medium [4,5]. Various methods were proposed in the literature for determining the minimum and maximum densities within the DEM framework [6,7]. Nevertheless, there is no standard procedure. A more straightforward approach is to compare it with the experimentally determined porosity or bulk density [8], especially if the grain shapes in the numerical model are close to the real ones [9]. The determination of porosity is simple if the particle density of the aggregate grains is known. Usually, this can be determined by water displacement methods (Archimedes’ method or graduated cylinders). However, in the case of a very porous material, such as expanded clay, the traditional approach carries the risk of making significant errors. Therefore, we are looking for alternative methods for measuring the volume and porosity of the expanded clay. We believe that the presented approach can also be applied to other porous aggregates, regardless of their origins (processed natural materials, waste materials, or naturally porous rocks, such as pumice, foamed lava, etc. [10]).

Structure from motion (SfM) is a photogrammetry-based framework for the 3D shape reconstruction of an object from images taken from different perspectives. Even though the reconstruction process is very complex, this framework has become very popular even among amateurs, thanks to the highly automated and user-friendly software that is freely available for use [11]. It is also extensively utilized in research studies across different fields and scales. On the one hand, it can be used in order to create large-area terrain elevation models (Wang et al., 2020), to document civil engineering structures [12] and archeological sites [13,14], or to study rock masses [15]. On the other hand, it can be used for relatively small volume measurements [16], the reconstruction of the shape of individual grains [17,18,19,20], or for studying the roughness of rock joints [21]. Especially, grain-scale measurements are particularly interesting in the context of the presented research. Paixão and colleagues proved that SfM is an accurate and convenient method of particle shape reconstruction [19]. They reconstructed relatively round and smooth particles (ballast grains after abrasion tests) and measured deviations between shapes obtained using SfM and laser scanning methods. Unfortunately, they scaled the SfM models in order to match the grains’ volume, which was measured with a laser scanner. Hence, the accuracy of the volume measurements cannot be concluded from the presented results. Ozturk and Rashidzade compared the shape descriptors computed based on the 3D and 2D imaging methods [18]. Five different types of granular materials were investigated, including one lightweight aggregate (perlite). They managed to obtain highly detailed models of aggregates. They also concluded that the proposed method is a cost-effective alternative to other 3D imaging techniques. However, a direct comparison to other 3D imaging techniques was not conducted. Recently, An et al. [17] proposed a framework for determining the particle shape and size by using smartphone photogrammetry. Within their simplified approach, multiple grains can be scanned and processed simultaneously. Nevertheless, the grains are not fully reconstructed, which makes it impossible to obtain accurate volume measurements. Zhao and colleagues [20] reconstructed the shapes of rock aggregates. Next, they generated particles of similar shapes within the DEM simulation and studied the relationship between the shape descriptors and the packing properties of the granular assembly (such as density, coordination number, etc.).

The main goal of our research is to accurately measure the particle density of LECA using the photogrammetry-based framework and compare the results with the ones obtained using computed tomography (CT). Compared with other grain-scale reconstructions, this approach focuses on the volume measurement and validation of the method by comparison with the CT technique. Moreover, the shape of lightweight aggregates differs significantly from typical rock materials used in civil engineering. They have irregular textures and are rich in asperities and holes (open pores) and these make it more challenging in order to measure the surface and to interpret the measurements in terms of volume. We will emphasize this problem in the methodology section. We believe that the outcome of our research can impact both engineers and researchers interested in utilizing lightweight aggregates. Our research can be applied to the following areas:

- Laboratory testing: the presented framework is a cost-effective method for measuring the particle density of porous aggregates (they do not require that the grains be immersed in a fluid that can penetrate the pores hence bias the result);
- Calibration of numerical simulations: popular calibration techniques of DEM models require assumptions about particle shapes and intergranular porosity [9]. Usually, the bulk volume and mass of the granular sample can be easily measured. However, the result of intergranular porosity depends on the grains’ particle density, which has to be measured as well.

## 2. Materials and Methods

### 2.1. Material

**Figure 1:**
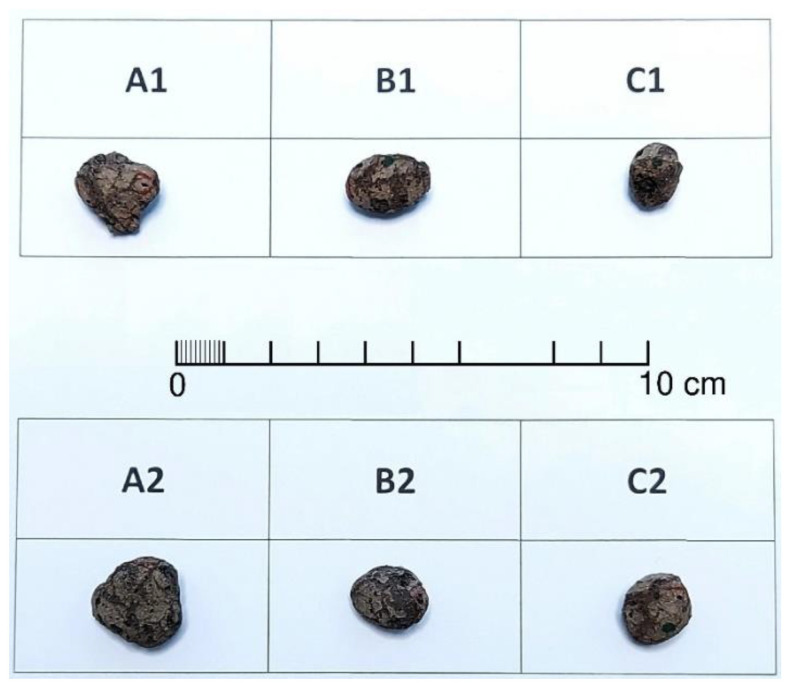
Six LECA grains selected for analysis: two subangular (**A1**,**A2**), two subrounded (**B1**,**B2**), and two rounded (**C1**,**C2**).

LECA^®^ that was produced in the Gniew plant in Poland, was used for this study. A 10–20 mm fraction was used, which is specifically utilized in geotechnical applications. This aggregate has a bulk density of 290–320 kg/m^3^. Based on the visual evaluation, three groups of aggregate shapes were distinguished (Figure 1).

The aggregates are denoted with the letters A, B, and C depending on the shape assessment: subangular, subrounded, and rounded, respectively (according to [22]). Two grains of each shape were selected for testing.

### 2.2. Computed Tomography Measurements

X-ray computed tomography, sometimes abbreviated as XCT or CT, uses X-ray radiation in order to take many two-dimensional (2D) images of an object in many positions, around an axis of rotation. From these images and using software, a three-dimensional (3D) model of the object’s external, as well as internal structure, are reconstructed and can be analyzed. The main reason for this method is for dimensional quality control purposes: i.e., for the traceable measurement and the tolerance verification of dimensions on mechanical components [23].

CT metrology is the only technology capable of measuring the inner and the outer geometries of a component without the need to cut through the component and destroy it. As such, it is the only technology used for the industrial quality control of workpieces having non-accessible internal features (e.g., components produced by additive manufacturing), multi-material components (e.g., two-component injection molded plastic parts or plastic parts with metallic inserts), or construction materials with a complex inner structure [24].

A bibliographical review about the use of the X-ray micro-tomography method for cementitious materials, microstructural investigations, for analyzing the microstructure of pores and their connectivity network, and for enabling the possibility of building a relationship between its permeability and porosity, is listed in [25]. The work clearly shows that this technique has enabled the understanding of the physical, chemical, and mechanical properties of cementitious materials, thus allowing for their further development. That is why we decided to use the CT method for the investigation of LECA in our research.

One should remember that CT scanners are of utmost importance if the internal structure of the device or material is to be investigated. The resolution can determine the smallest detail (step or feature) that is still perceptible or inducible within a machine: e.g., the smallest displacement or change of length that can be identified on an analog or digital readout scale or the smallest inclusion that is still perceptible on an optical or X-ray image sensor. However, the only characteristic describing the quality of a specific measurement result is the measurement uncertainty.

The LECA grain measurements were performed using the computed tomography system METROTOM 800 by Carl Zeiss (Oberkochen, Germany), which is located in the Institute of Metrology and Biomedical Engineering laboratory of the Faculty of Mechatronics of Warsaw University of Technology. The computed tomography system METROTOM 800 is equipped with a transmission X-ray tube with a power of 39 W (at maximum parameters—130 kV voltage and 300 μA current) and a detector with a resolution of 1536 × 1920 pixels and 127 µm pixel size. The MPE_E_ (Maximum Permissible Error, Error of indication), according to VDI/VDE 2630, is 4.5 + L/100 μm, where L is the measured length in mm. The measurement parameters have been set in the METROTOM OS software (V3.2, Carl Zeiss, Oberkochen, Germany). A summary of measurement parameters is presented in Table 1.

One can note that the voxel size in the current setup of CT measurements is approximately 34 µm. Therefore, we are capable of capturing only relatively large macropores. This aligns with our goals since we are interested in computing the envelope volume of particles and we have disregarded the information about the porosity during the analysis.

### 2.3. Remarks on CT Volume Measurement

**Figure 2:**
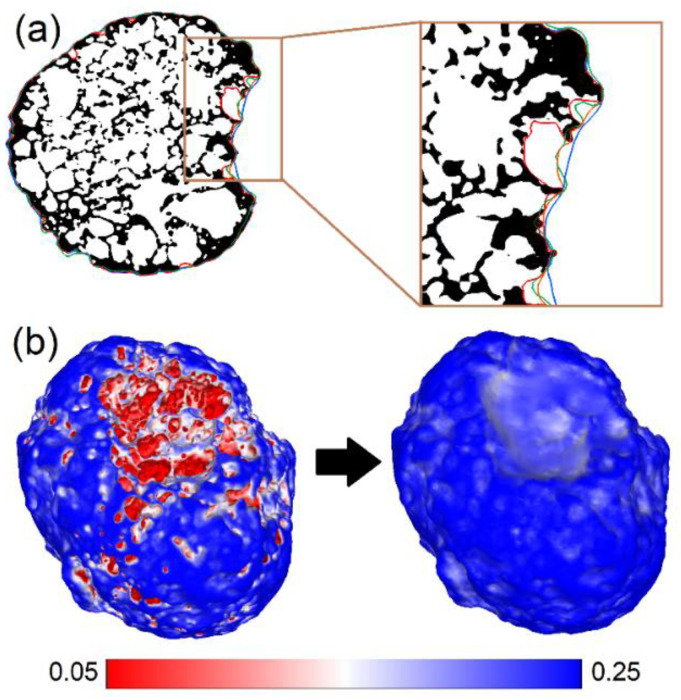
Influence of the Ambient Occlusion threshold on the determined CT model’s envelope: (**a**) Cross-section of grain C1’s full CT model, (**b**) External surface of grain C1 obtained after the simplification with the threshold t=0.05 (**left**) and t=0.175 (**right**).

Computed tomography is an imaging technique that allows for an object’s precise 3D reconstruction, including its internal structure. The tested LECA grains are composed of a highly porous material. A cross-section example of a grain’s 3D model is presented in Figure 2a. While estimating the particle density, we are interested in the total volume of the grains (including pores). However, many pores are open, making it difficult to decide if they should be excluded from the grain volume or not. Furthermore, we need a procedure in order to subtract what we define as the external surface (envelope) of the grain. The Ambient Occlusion algorithm implemented in the MeshLab software [26] covers the technical side of this problem. The algorithm ‘looks’ at the 3-dimensional grain reconstruction from different perspectives (120 views in our setup) and counts how often a certain point of the mesh is visible. The internal surfaces of the closed pores are always hidden. Most of the crust surface is usually visible. However, the chances of capturing the points on the surface of the cavities (such as open pores) are small and depend on the grain geometry and the number of grain views. One can see an analogy with the photogrammetric reconstruction, where the point position can only be estimated if it is visible on at least two images. The Ambient Occlusion algorithm stores the information about the visibility frequency for each point. Next, the mesh subset can be selected by applying the threshold t. The lower the threshold we choose, the deeper we look into the cavities in order to extract the external surface of the grain. The selected subset surface always contains a number of holes. Therefore, the final version of the modeled grain needs to be reconstructed. We utilized the *Screened Poisson Surface Reconstruction* algorithm [27] with parameters listed in Section 2.4.3. In this way, we obtained a *close-fitting imaginary envelope* that contained the whole grain and cut off the open pores from grain’s surroundings. Hence, the volume that we obtain is called the envelope volume, according to ASTM D3766 [28,29], and consequently, the particle density can also be called the envelope density.

Since the threshold t parameter may affect the results, its influence was evaluated by preparing grain models with different levels of t (0.05, 0.075, 0.100, 0.125, 0.150, and 0.175). Figure 2b shows the surface of grain C1 reconstructed with different values selected for the threshold t=0.05 (left) and t=0.175 (right). Furthermore, cross-sections of alternative surface reconstructions are presented in Figure 2a (red for t=0.05, green for t=0.125, and blue for t=0.175). The orange line shows the cross-section of the surface obtained from the SfM algorithm for comparison.

### 2.4. Photogrammetry Workflow

Generally, the SfM workflow consists of the following main steps: photographing the physical object from different angles, the automatic recognition of characteristic features in different pictures, computing the relative camera positions in the common coordinate system, the computation of the spatial coordinates of the object’s portions that are visible in the images, and postprocessing (including cleaning, scaling, and textured mesh reconstruction). The approach adopted in our work is similar to the one presented by other authors dealing with rock grains. A number of them decided to glue the grain onto a thin pedestal, which allowed them to photograph almost the whole surface of the grain in one session [18,19]. In contrast, other authors placed the grains onto a flat surface, which resulted in an incomplete grain reconstruction that required fixing later during the postprocessing stage [17,20]. We aimed to measure the volume as accurately as possible with minimum intervention to the grain’s structure. At the same time, we wanted to keep the acquisition process as simple as possible. Thus, we decided to split the reconstructed grains into two later merged models. Each grain was photographed in the first session, then flipped 180 degrees, and photographed again. The details of the process are described below.

#### 2.4.1. Sample and Scene Preparation

**Figure 3:**
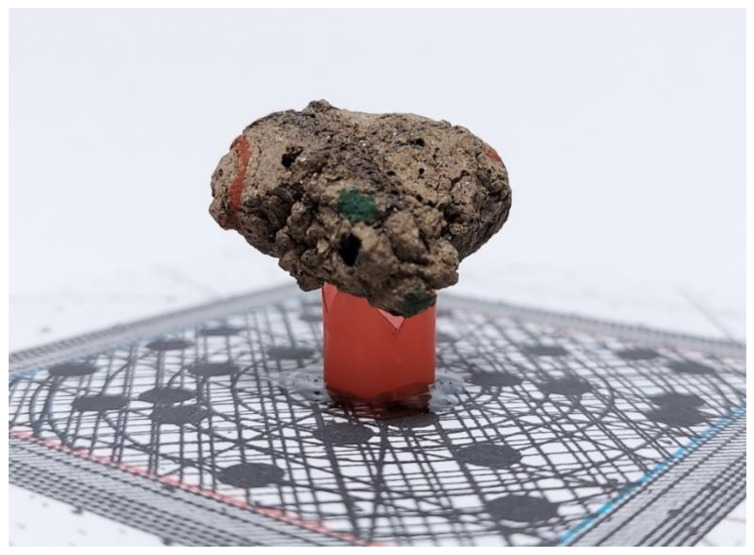
LECA prepared for image acquisition.

The acquisition scene was prepared on an office desk with no additional light source (only good-quality ceiling lamps). The background consisted of a sheet of white paper. It allowed tricking the SfM algorithm and to rotate the grain on its stand instead of moving the camera around the object [19]. The grain support consisted of a plastic straw glued onto the top of a small box (10 cm × 10 cm × 2 cm). The straw was relatively wide and saw-edged. Hence, it provided a stable support without the need for gluing the grain. A printed pattern was placed in between the top of the box and the straw. The pattern consisted of two components: an irregular arrangement of lines and dots that was supposed to provide additional features for the SfM algorithm and the square of the dimensions 40 mm × 40 mm that were later used for the scale computation. A photograph of the grain prepared for the image acquisition is presented in Figure 3. A picture of a segmented circle was placed beneath the box. It was used for the manual control of the box rotation.

#### 2.4.2. Image Acquisition

We acquired images with a smartphone (Samsung Galaxy S10 Lite, Suwon-si, South Korea) camera (SM-G770U1) and worked in the manual mode with the following settings: resolution 3000 × 4000 px, f/2 aperture, 0.125 s exposure time, and ISO 50. It allowed us to obtain bright and sharp photos despite no additional light sources (except for ceiling lamps).

Once the grain was positioned in place, two pictures were taken from a distance of 6–7 cm. The first picture was taken with the camera at the same height as the grain. In the second configuration, the camera was positioned higher than the grain and oriented at an angle of approximately 45 degrees to the ground. Next, the bottom box with the grain holder was manually rotated 10 degrees, and another two pictures were taken. This procedure was repeated until the grain was fully rotated. Thus, 72 pictures were acquired for each model. Although, some pictures were discarded due to quality issues, thereby resulting in 60–72 photos for every reconstruction. This is a minimal configuration that allows to fully reconstruct the grain’s upper half. Next, we flipped the grain 180 degrees and repeated the acquisition procedure. Thus, every grain was photographed up to 144 times and reconstructed into two separate models.

#### 2.4.3. 3D Modeling and Postprocessing

There are many alternatives for free SfM software that allow for cloud, desktop, or even smartphone-based processing [11]. We selected MVE (Multi-View Environment developed by Simon Fuhrmann and others in TU Darmstadt, Germany) [30] because of its low hardware requirements, thereby making it accessible to more potential users. For example, a CUDA-compatible graphics card is not required. The reconstruction was conducted according to the typical workflow described in [31]. The reconstruction outcome consisted of textured meshes of the photographed scenes.

Further postprocessing was conducted using Meshlab [26]. First, the poor-quality faces and vertices were filtered out. In the next step, small, disconnected components were removed. At this stage, the scene contained both the grain and bottom box with the printed pattern. The length of the four sides of the printed square was measured manually and averaged. Based on this information, the whole model was scaled uniformly in order to match the actual dimensions of the pattern. Next, the box and grain holder were removed from the mesh. The rough manual selection was sufficient because the grain surface can be partially removed as well since the missing portion of the grain is to be replaced by the surface from the second reconstruction. Hence, two meshes were prepared for every grain, each representing 60–70% of its surface. It allowed to manually align the meshes based on the corresponding points from both meshes. The color pattern that was applied to the grains before the image acquisition was visible on the textured meshes, making it easier to find the corresponding points. Following the rough alignment, the position of the mesh was adjusted by minimizing the distance between the overlapping portions of the surfaces. The two aligned meshes were merged into one ‘watertight’ mesh using the *Screened Poisson Surface Reconstruction* algorithm [27] implemented in Meshlab. The parameters selected for the algorithm were as follows: *reconstruction depth*: 12; *minimum number of samples*: 8; *interpolation weight*: 2; *pre-clean*: “on”.

#### 2.4.4. Comparison of SfM and CT Models

The validation of the volume measurement method based on the SfM reconstruction is crucial in order to evaluate its applicability for the estimation of the particle density. The outcome of the above-described 3D modeling procedure is the watertight meshes of the grain surfaces. The volume of such meshes can easily be computed and compared with the simplified grain models obtained from the CT measurements. By simplified model, we understand the extracted external envelope of the grain, according to the procedure described in Section 2.3. Nevertheless, the value of the threshold parameter t selected for the interpretation of the outer grain envelope may significantly influence the computed volume (see Figure 2). The photogrammetry-based models were subjected to the same simplification procedure as the CT measurements (with t=0.175) in order to minimize the influence of this threshold. The volumes of the simplified and non-simplified models are compared using these two methods.

Additionally, the accuracy of the 3D shape reconstruction is estimated. First, two corresponding meshes are aligned (the same grain, following the CT and SfM reconstructions with t=0.175). Next, the Hausdorff distance [32] is computed between the two surfaces using the Meshlab functionality.

#### 2.4.5. Archimedes’ Method

**Figure 4:**
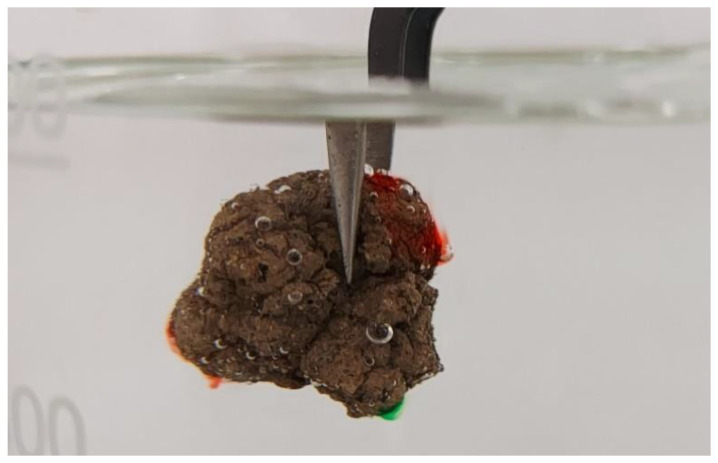
LECA grain immersed in water.

Archimedes’ method is a traditional method for measuring the grain volume. This requires immersing the grain in water. In the case of porous materials, this method has some drawbacks. On the one hand, the surface of the LECA grain is rough, which causes air bubbles to adhere (the bubbles can be removed but it takes time). On the other hand, the sample should not be immersed in water for a long period of time because the water infiltrates the pores and lowers the result of the volume measurement (see Figure 4). Nevertheless, we compare the final results of the density measurement with the results using a traditional approach.

In Figure 4, one can see a grain immersed in water. Because the density of the LECA grain is smaller than water, the grain needs to be pushed down. This is achieved with stabilizing tweezers in the stand (frame). Since the frame rests on a bench, the measured weight change equals the weight of the displaced water. The grain is immersed for a short period of time (up to 15 s).

## 3. Results and Discussion

The most interesting result, in terms of the particle density measurement, is the volume estimation. The results obtained from both methods are presented in Table 2. One can see that the volume estimated from the CT models increases as we increase the threshold *t*. It is expected because, for higher values of the threshold, we include more open pores into the total volume of the grain.

If we compare the CT and SfM (without thresholding), we can see that in most cases (except for the grain A2), the SfM results fall within the range of the CT-based volume estimation. Analyzing the same data, one can notice that in the case of four grains, the SfM-based evaluation gives smaller values than the CT for t=0.125. We conclude that the average capability of the SfM surface reconstruction (in terms of capturing the surface of open pores) corresponds to the CT measurement that was post-processed with this value of the threshold applied (compare with Figure 2a). Of course, this conclusion applies to the models obtained for certain acquisition settings. It is likely that the pore surfaces could be better reconstructed if more images are used for the reconstructions.

**Figure 5:**
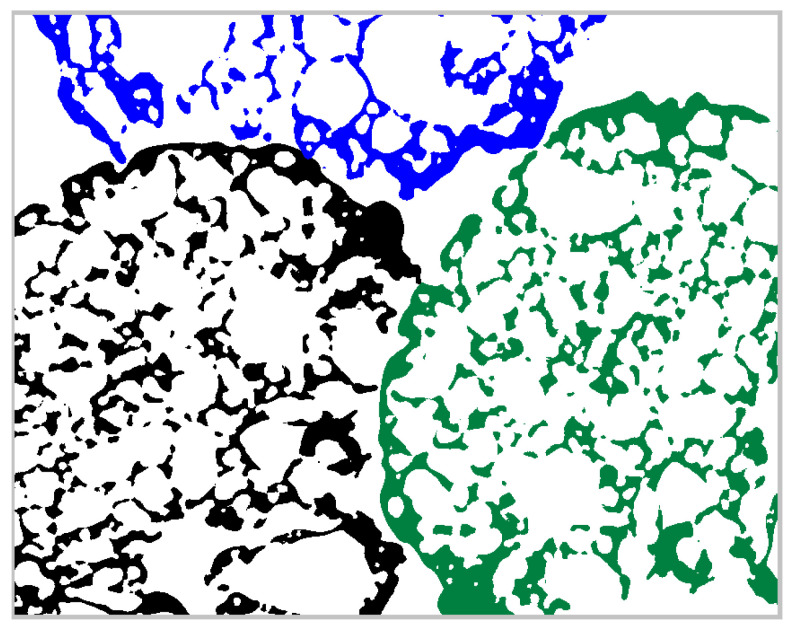
Visualization of the potential dense packing of three particles.

In order to exclude the influence of the thresholding, we decided to compare the volumes of models post-processed with the same threshold value (t=0.175). It is an open question whether this is the best choice for the particle density estimation. Surely, the answer depends on the target application. For example, in the calibration of the DEM models, we are interested in the assessment of the intergranular porosity of the granular assembly. Therefore, the grain envelope should involve open pores that are unlikely to be penetrated by the asperities of the other grains. Yet, the wide and shallow cavities can be easily filled by the rounded shapes of the neighbor particles. This is visualized in Figure 5.

In Figure 5, one can note that a wide shallow cavity of the black-colored grain can be filled by the green grain. However, the further filling of the open pores is very unlikely. Therefore, we ignore the relatively deep cavities in the analysis by applying the threshold t=0.175 (the envelope obtained with this threshold is presented in Figure 2).

A comparison of the particle density estimation based on the SfM, Archimedes’ method, and CT volume measurements is presented in Table 3.

The error of the SfM volume estimation (taking CT with t=0.175 as reference) ranges from −0.97% to 1.56%, with a mean value of −0.33% (0.0016 g/cm^3^). Since we have one set of grains measured using different methods, we can compare the results with the paired sample *t*-Test. In a two-sided test with a significance level α=0.1, we cannot reject the null hypothesis. In other words, the difference between the CT and SfM methods is statistically insignificant. The error of Archimedes’ method ranges between 1.39% and 3.59%, with a mean value of 2.49% (0.0120 g/cm^3^). One can note that this method always slightly overestimates the density measurement (due to the water quickly penetrating the open pores during measurement). Thus, the observed difference is statistically significant.

The ‘quality’ of such a conclusion can depend on many factors, including on the number of grains measured. One can rearrange the problem and ask how sensitive this statistical test is for the conducted experiment. We verified this with a solver implemented in the statsmodels Python package [33]. The difference that can be detected with a power of 0.8 is equal to 0.006 g/cm^3^. In other words, if the SfM measurement introduced an error greater than 0.006 g/cm^3^, the test would classify this difference as significant. This is an acceptable accuracy, proving that the proposed framework for the particle density estimation of porous aggregates is valid.

**Figure 6:**
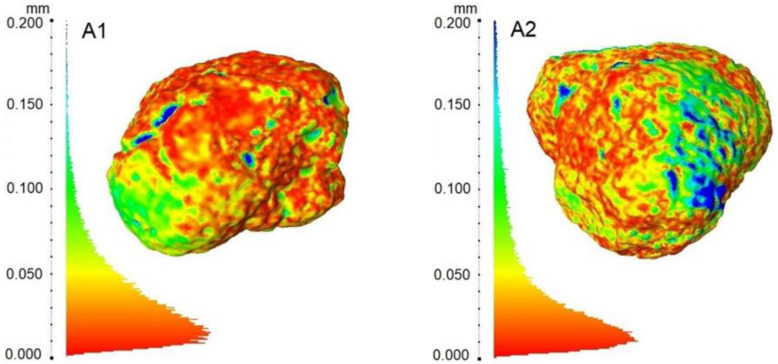

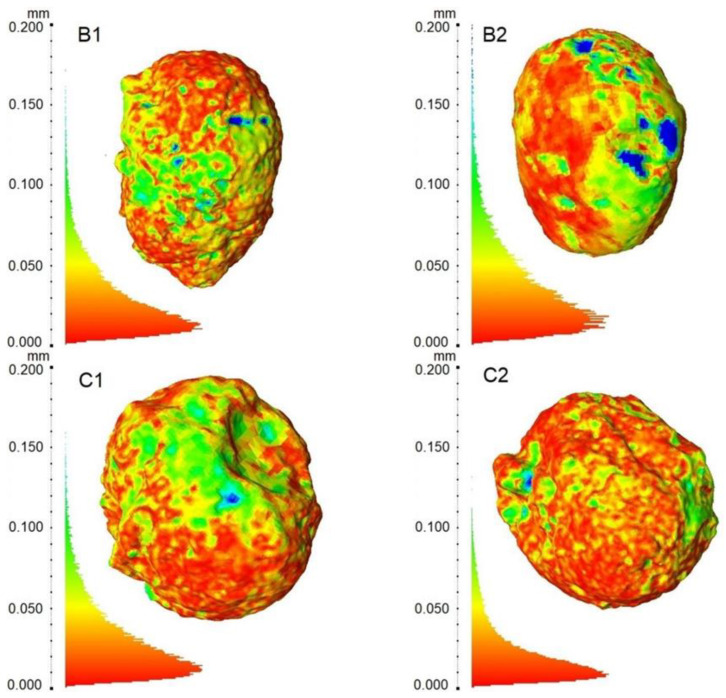
Difference between the CT-based and photogrammetry-based models measured as the Hausdorff distance (area-weighted histograms and error distribution on the grain surfaces) for samples (**A1**–**C2**).

We conducted further analysis in order to evaluate the shape reconstruction quality by measuring the Hausdorff distance (error) between the aligned SfM and CT models of the grains. The area-weighted histograms of the computed distance are presented in Figure 6, along with color maps of the error distribution on the grain surfaces. Further statistics of the surface discrepancies are summarized in Table 4.

From Figure 6, one can see that the highest error areas are concentrated in small spots (blue dots). Despite the unified postprocessing method, the small open pores can still be the source of error with a relatively large magnitude (greater than 0.15 mm). Nevertheless, their small area limits the impact on the volume measurement. The differences concerning the smaller amplitude (0.05–0.015 mm) can be observed to a greater extent and do not seem to correlate with the characteristic features of the grains.

The statistics summarized in Table 4 confirm that even though the maximum errors reach 0.210–0.485 mm, most of the surface (95%) is reconstructed with a greater accuracy than 0.065–0.129 mm. One can also notice that any error seems to be dependent upon the shape of the particle. The particles with the most complex, subangular shapes (A1 and A2) are represented with the least amount of accuracy, while the particles classified as rounded (C1 and C2) are the best reproduced. Furthermore, it should be emphasized that the magnitude of the mean and RMS (Root Mean Square) of errors are comparable with the estimated resolution of the CT scans (0.045 mm). Hence, it is difficult to distinguish how much both methods contribute to the measured discrepancy. It shows that photogrammetry allows for very accurate surface measurements at a low cost.

## 4. Conclusions

The paper addresses issues related to the measurement of the particle density of LECA using a photogrammetry-based volume measurement framework. Traditional volume measurement methods require that a sample be immersed in water. Nevertheless, water penetrates the pores of the LECA grains, affecting the results [2]. Computed tomography is another alternative. This method allows for very accurate shape measurements (including of internal structures). Hence, we chose this as a reference for the validation of the proposed photogrammetric approach. Based on the conducted analysis, the following conclusions can be drawn:

- In the case of aggregates with open pores, such as LECA, the particle volume (envelope volume) estimation depends on the envelope definition;
- We proposed a procedure for the envelope computation that can be applied to 3D models obtained from CT and SfM measurements;
- We recommend applying the threshold (t) of 0.175 for the grain envelope computation if the method is used in order to assess the intergranular porosity of LECA;
- We compared the particle density of grains obtained by the different methods. The SfM approach underestimated the mean density by 0.33% (0.0016 g/cm^3^), while Archimedes’ method overestimated the density by 2.49% (0.0120 g/cm^3^). All three methods show sufficient accuracy for most engineering purposes. Nevertheless, the SfM is the most accurate;
- The shape reconstruction with the SfM approach is very accurate. For 95% of the grain surface, the error is no higher than 0.129 mm (in the case of irregularly shaped grains) and no higher than 0.073 mm (in the case of round grains).

The presented approach can be utilized for measuring the particle density of porous aggregates. This information can be used in order to estimate the intergranular porosity, which in turn is valuable information for the calibration of DEM models.

## Figures and Tables

**Table 1:** Summary of CT measurement parameters.

Parameter	Value
X-ray tube voltage	80 kV
X-ray tube current	120 µA
Integration time	800 ms
Detector gain	8×
Number of projections	1500
Voxel size	34 µm
Focal spot control	YES–Frame interval 64
Noise reduction filter	Shepp Logan

**Table 2:** Volume of grains computed with different methods and different thresholds applied.

Grain	Computed Volume (mm^3^)						
	CT Model					SFM Model	
	*t* = 0.050	*t* = 0.075	*t* = 0.100	*t* = 0.125	*t* = 0.150	*t* = 0.175	No Threshold
A1	1340.39	1346.89	1354.07	1362.07	1370.14	1381.08	1345.96
A2	2167.83	2177.34	2185.38	2191.19	2196.73	2202.93	2210.19
B1	1315.02	1318.21	1320.74	1323.69	1327.25	1331.44	1321.53
B2	1249.45	1255.90	1260.36	1264.08	1270.02	1275.76	1257.78
C1	774.25	779.20	784.81	789.74	793.94	798.85	784.93
C2	1409.53	1415.49	1417.71	1420.44	1424.19	1429.29	1426.71

**Table 3:** Particle density results obtained with the photogrammetry-based (SfM), laboratory (Archimedes’ method), and computed tomography volume measurements.

Grain	Mass (g)		Density (g/cm^3^)		Error (%)	
		SfM	Archimedes’	CT	SfM	Archimedes’
A1	0.66390	0.4883	0.4979	0.4807	1.59%	3.59%
A2	1.03283	0.4643	0.4814	0.4688	−0.97%	2.69%
B1	0.57681	0.4350	0.4392	0.4332	0.40%	1.39%
B2	0.68286	0.5383	0.5449	0.5353	0.56%	1.81%
C1	0.36727	0.4624	0.4697	0.4597	0.58%	2.16%
C2	0.72321	0.5051	0.5228	0.5060	−0.18%	3.32%
	mean	0.4822	0.4927	0.4806	0.33%	2.49%

**Table 4:** Error of the surface measurement (Hausdorff distance from the photogrammetry-reconstructed surface to the CT model surface).

Grain	Reconstruction Error (mm)			
	Max	Mean	RMS	95th Percentile
A1	0.397	0.038	0.052	0.102
A2	0.485	0.048	0.067	0.129
B1	0.347	0.029	0.040	0.080
B2	0.448	0.038	0.053	0.105
C1	0.247	0.027	0.036	0.073
C2	0.210	0.021	0.030	0.065

## Data Availability

Not applicable.
